# Mixed small cell neuroendocrine carcinoma and squamous cell carcinoma covered by tubulovillous adenoma in the rectum: a case report and detailed molecular analyses

**DOI:** 10.1186/s12957-023-02954-6

**Published:** 2023-02-27

**Authors:** Fengxia He, Xue He, Menghan Cui, Yan Wang

**Affiliations:** Department of Pathology, the Second Affiliated Hospital of Nanjing Medical University, Jiangjiayuan 121#, Nanjing, 210011 Jiangsu Province China

**Keywords:** Mixed neuroendocrine-non-neuroendocrine neoplasm (MiNEN), Squamous cell carcinoma, Rectum tumor, Next-generation sequencing (NGS)

## Abstract

**Background:**

Previously, only six cases of mixed neuroendocrine-non-neuroendocrine neoplasm (MiNENs) with squamous cell carcinoma (SCC) component have been described in the colorectum, and the molecular landscape of MiNENs is also poorly understood. Herein, we present a unique case in which the SCC developed as a component of a MiNEN in the rectum.

**Case presentation:**

The patient was firstly diagnosed as rectal small cell neuroendocrine carcinoma (SCNEC) covered by tubulovillous adenoma, and then mixed SCNEC and SCC in the same site 6 months later. Representative samples from the three histologic subtypes were then sent for next-generation sequencing (NGS) separately. Multiple liver metastases occurred in the following month after the last surgery. The patient died of ketoacidosis 1 year after initial diagnosis of the tumor.

**Conclusion:**

This is the first report of this exceedingly rare tumor type to include NGS of the 3 separate morphological entities. Our findings may expedite the understanding of combined tumors in the colorectum.

## Background

Neuroendocrine carcinomas (NEC) are highly aggressive tumors with poor clinical outcome, accounting for < 1% of all tumors of the colorectum [1]. Squamous cell carcinomas (SCC), which usually involve the esophagus or anal canal, are even more rare in the colorectum [2]. Tumors consisting of NEC and SCC, which belong to mixed neuroendocrine-non-neuroendocrine neoplasms (MiNENs), mostly also occur in above-mentioned organs with original squamous epithelium [3]. Herein, we present a unique case of a patient who was firstly diagnosed as rectal small cell NEC (SCNEC) covered by tubulovillous adenoma, and then mixed SCNEC and SCC in the same site 6 months later, of whom multiple liver metastases developed shortly after the last surgery.

## Case presentation

**Fig. 1:**
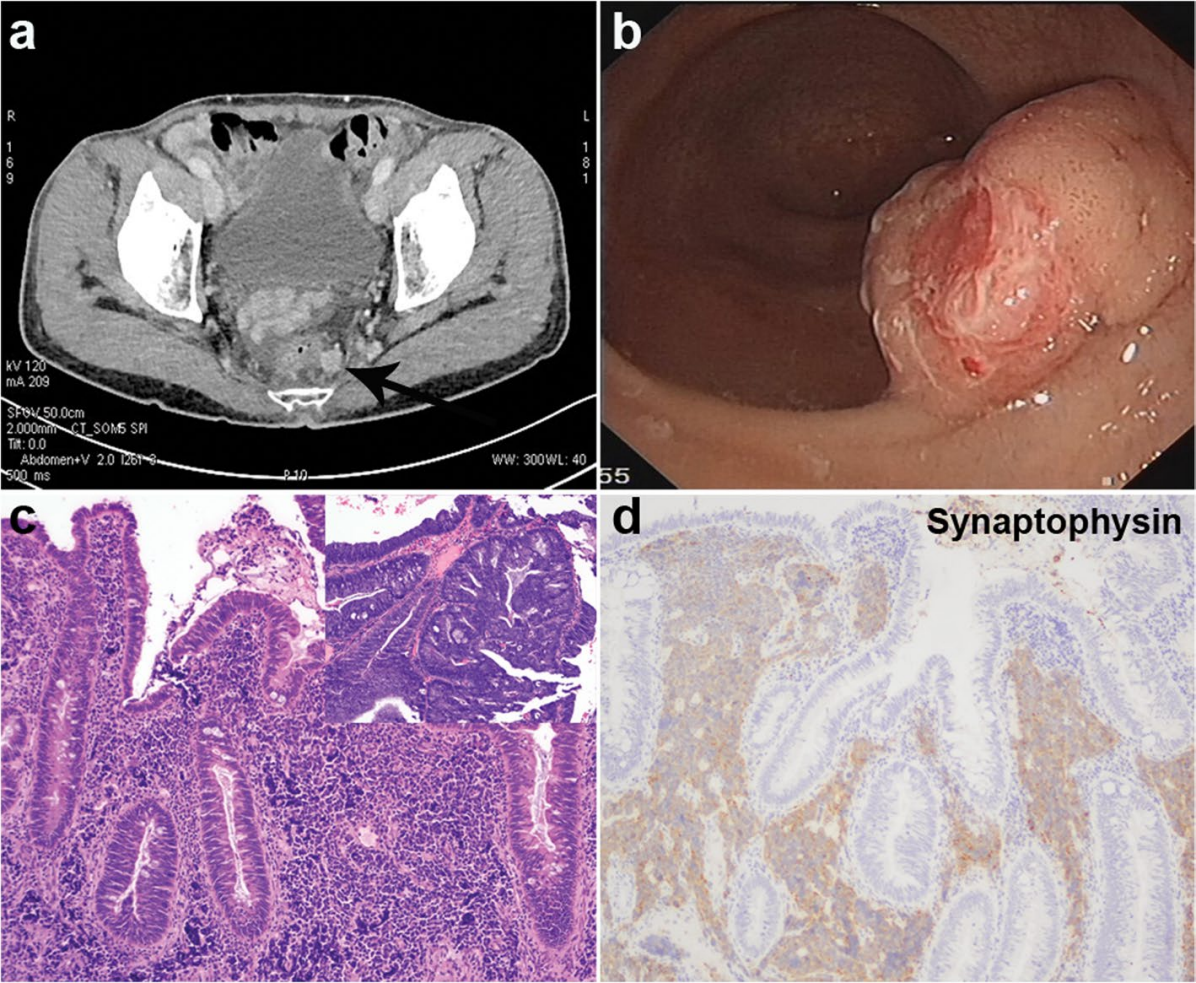
Representative images of the first operation. **a** CT of the abdomen revealed thickening rectal wall and swollen lymph nodes around the rectum. **b** Colonoscopy showed a 2.0 cm × 3.0 cm irregular bulge in the rectum. **c** The tumor exhibited a tubulovillous adenoma with high-grade dysplasia (upper right) on the surface and a component of SCNEC limited to the submucosa. **d** SCNEC was positive for synaptophysin

A 71-year-old male patient without relevant pre-existing conditions was admitted to our hospital due to altered bowel habit and hematochezia. Abdomen computed tomography (CT) revealed rectal wall thickening and swollen lymph nodes around the rectum (Fig. 1a). Tumor markers were within normal range. Subsequent colonoscopy showed a 2.0 cm × 3.0 cm irregular uplifted neoplasm in the rectum 3 cm from the anal verge (Fig. 1b). Transanal endoscopic microsurgery (TEM) [4] instead of Miles surgery was then performed at the urging of the patient. Microscopic analysis (Fig. 1c, d) exhibited a tubulovillous adenoma with high-grade dysplasia on the surface, and a component of poorly differentiated SCNEC with typical morphological and immunohistochemical characteristics (positive for synaptophysin and CD56, but negative for chromogranin A) limited to the submucosa. Resection margins were free of tumor cells. No chemotherapy was performed after the surgery.

**Fig. 2:**
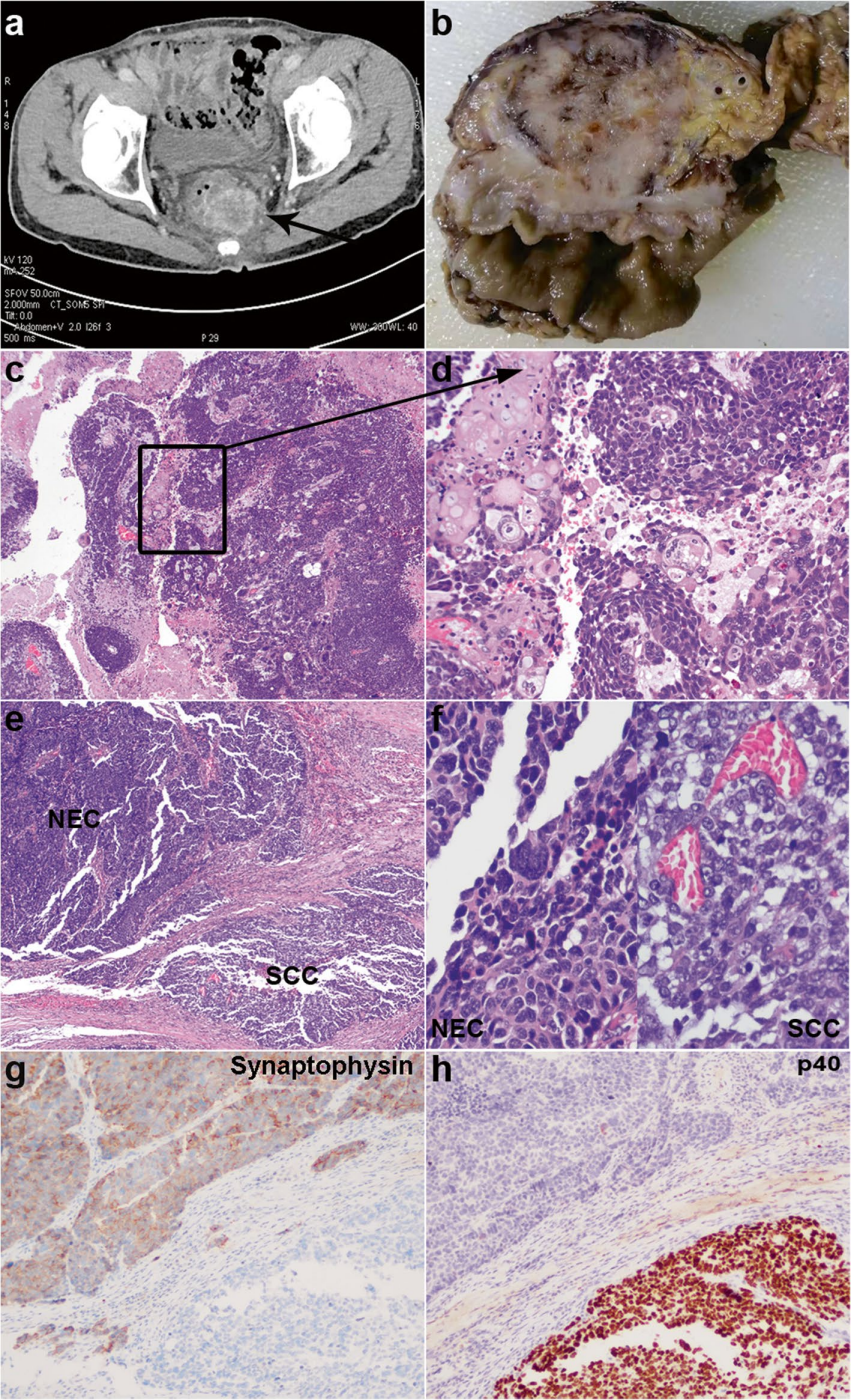
Representative images of the last operation. **a** Abdomen CT showed more obvious rectal wall thickening and an inhomogeneous enhanced mass on the left side of rectum. **b** Transverse section of the mass demonstrated a tan-white to gray cut surface, and the surrounding adipose tissue was involved. **c** A solid and perivascular papillary growth pattern with extensive necrosis at low magnification. **d** Prominent squamous appearance with keratin pearl formation at higher magnification, and tumor giant cells were remarkable. **e** Low power field which contains both SCC (lower right) and NEC (upper left). **f** Higher magnification of NEC (left) and SCC (right). NEC showed a scant basophilic cytoplasm, “salt and pepper” chromatin, and inconspicuous nucleoli. SCC displayed a faintly stained cytoplasm and prominent nucleoli. Synaptophysin (**g**) was exclusively present in NEC, and p40 (**h**) was exclusively present in SCC

The patient was hospitalized again with hematochezia 6 months later. Abdomen CT showed an inhomogeneous enhanced mass on the left side of rectum which was more pronounced than before (Fig. 2a). Colonoscopy showed a bulge of 1.5 cm in diameter near the anastomotic stoma. Then, Dixon operation was carried out. Macroscopically (Fig. 2b), the mass had a tan-white to gray cut surface and invaded the perirectal adipose tissue. Microscopically (Fig. 2c–h), a solid and perivascular papillary growth pattern with extensive necrosis was observed. Most areas showed distinct squamous appearance with keratin pearl formation and immunohistochemical expression of p40, but not of synaptophysin. Meanwhile, clusters of tumor cells, which were characterized by scant basophilic cytoplasm and “salt and pepper” chromatin, were positive for synaptophysin but negative for p40. Ki-67 index was ~40% and ~90% for SCC and SCNEC, respectively. All tumor cells exhibited positive expression of β-catenin (cytoplasmic and nuclear) and CDX2. SATB2 was positive in SCNEC but not in SCC. In summary, a mixed SCNEC and SCC of the rectum was diagnosed, and only one regional lymph node metastasis with SCNEC was detected.

CT showed multiple liver metastases in the following month. The patient was discharged from the hospital before his first cycle of chemotherapy (etoposide, oxaliplatin plus bevacizumab) was completed. Poor appetite and elevated serum NSE level were observed during follow-up. The patient died of ketoacidosis 1 year after initial diagnosis of the tumor.

## Discussion

Previously, only six cases of MiNENs with SCC component have been described in the colorectum [1, 5–8], and only one tumor was histologically covered by tubulovillous adenoma like ours [7]. It is currently hypothesized that the adenocarcinoma component and NEC component of mixed adenoneuroendocrine carcinoma (MANEC, a subgroup of MiNEN [3]) arise from either multipotent stem cells or 2 similar but separate precursors. However, the pathogenesis of mixed SCC and NEC is not well-defined. Possible causes for squamous colonic carcinoma are malignant transformation of persistent ectopic embryonal nests of ectodermal cells, chronic inflammation, human papillomavirus, pelvic radiation exposure, etc. [1, 2]. Some authors postulated that the non-neuroendocrine component might give rise to a neuroendocrine component through the trans-differentiation process and then acquire a more aggressive phenotype [9]. Molecular analysis was performed in three of the previous six cases, including next-generation sequencing (NGS) in two cases [1]. Nevertheless, neuroendocrine and squamous components were not micro-dissected separately.

In present case, the representative samples from each histologic subtype were sent for NGS separately. Sequence data were evaluated (Table 1) included point mutations, fragment insertion and deletion, gene fusions/rearrangements, copy number alterations, microsatellite state, and tumor mutation burden (TMB). The tumor harbored multiple alterations in genes that are established drivers of oncogenesis, with APC and TP53 mutations being the most frequently found. Other genes, like KRAS, BRCA1, FBXW7, and RNF43, were also found to be altered. All of these mutations shared in all three lineages, supporting the hypothesis of their common clonal origin. However, there were also alterations exclusively present in one or two of the three components, such as RB1 frameshift mutation in SCC and NEC, high copy number gain of CDK4 in NEC, and KMT2B missense mutation in SCC, suggesting that at some point in the tumorigenic process, distinct morphological entities emerge through the activation of separate genetic programs. In addition, missense mutations of FLT4, MLH3, PDGFRB, and PKHD1, of which the specific significances were unknown, were presented in all three histological types as well. Moreover, TMB was high in SCC but low in NEC and adenoma, and the tumors were devoid of microsatellite instability without exception.

**Table 1:** Results of next-generation sequencing analysis

Gene	Mutations	Abundance (%)		
		Adenoma	NEC	SCC
KRAS	c.436G>A (p.A146T)	25.5	28.3	31.7
BRCA1	c.4987-2A>G	45.3	34.2	27.7
CDK4	Copy number amplification	-	CN: 4.6	-
TP53	c.159G>A (p.W53*)	41.6	85.2	79.8
APC	c.3964G>T (p.E1322*)	52.3	94.0	78.3
FBXW7	c.217C>T (p.Q73*)	27.6	47.5	37.3
FBXW7	c.372+1G>A	26.4	44.2	32.4
RB1	c.1963dup (p.Y655Lfs*13)	-	67.3%	71.2%
RNF43	c.131del (p.Q44Rfs*7)	25.5	52.1	53.6
FLT4	c.1426C>T (p.R476W)	28.9	49.6	52.5
KMT2B	c.74G>A (p.R25Q)	-	-	22.7
MLH3	c.484A>G (p.M162V)	49.4	7.3	14.3
PDGFRB	c.886A>T (p.S296C)	22.2	6.2	8.8
PKHD1	c.11006C>T (p.S3669L)	26.3	60.9	53.1
TMB	Mutations/Mbases	6.2	8.2	10.3
	Low < 10	Low	Low	High
	High ≥10			
Microsatellite		Stable	Stable	Stable

Our case highlights a rare disease, in which a SCC developed as a component of a MiNEN in the colorectum, and this is the first report of this exceedingly rare tumor type to include NGS of the 3 separate morphological entities. TP53, APC, and KRAS mutation may be a “trunk,” as they were presented in all tumor clones and were likely involved in driving the tumorigenic process. Other genetic alterations involved may be potential mediators of the trans-differentiation process. We suppose that the tumor might not have been completely removed at the first TEM operation, and lymph node dissection and postoperative chemotherapy were not performed, thus leading to a rapid relapse. In addition, the stimulation of the original surgery might lead to a squamous cell transformation.

Our findings may expedite the understanding of combined tumors in the colorectum. Further research, especially with regard to divergent differentiation of neuroendocrine- and squamous-related genes, is necessary to fully decode the development of this mixed neoplasm.

## Data Availability

Not applicable.
